# Crystal structure of (2,2′-bi­pyridine-κ^2^ *N*,*N*′)bis­(3,5-di-*tert*-butyl-*o*-benzo­quinonato-κ^2^ *O*,*O*′)ruthenium(II)

**DOI:** 10.1107/S205698901700281X

**Published:** 2017-02-28

**Authors:** Md. Serajul Haque Faizi, Akram Ali, Vadim A. Potaskalov

**Affiliations:** a Department of Chemistry, College of Science, Sultan Qaboos University, PO Box 36, Al-Khod 123, Muscat, Sultanate of Oman; b Department of Chemistry, Indian Institute of Technology, Kanpur 208 016 UP, India; c Department of General and Inorganic Chemistry, National Technical University of Ukraine, Kyiv Polytechnic Institute, 37 Prospect Peremogy, 03056 Kiev, Ukraine

**Keywords:** crystal structure, Ru(II) complex, π–π stacking, 3,5-di-*tert*-butyl-*o-*benzo­quinone, 2,2′-bi­pyridine

## Abstract

In the title mononuclear complex, [Ru(C_14_H_20_O_2_)_2_(C_10_H_8_N_2_)], the Ru^II^ ion has a distorted octa­hedral coordination environment defined by two N atoms of the chelating 2,2′-bi­pyridine ligand and four O atoms from two 3,5-di-*tert*-butyl-*o-*benzo­quinone ligands. In the crystal, the complex mol­ecules are linked by inter­molecular C—H⋯O hydrogen bonds and π–π stacking inter­actions between the 2,2′-bi­pyridine ligands [centroid–centroid distance = 3.538 (3) Å], resulting in a layer structure extending parallel to the *ab* plane.

## Abstract 2

In the title compound, the Ru^II^ ion has a distorted octa­hedral RuN_2_O_4_ coordination environment defined by two 3,5-di-*tert*-butyl-*o-*benzosemi­quinone ligands and one 2,2′-bi­pyridine ligand. In the crystal, the compounds are linked by C—H⋯O and π–π stacking inter­actions, resulting in a layer structure.

## Chemical context   

The coordination chemistry of *o*-quinone ligands has been a subject of inter­est since the beginning of the century, but only within the past decade have detailed studies on the composition and properties of *o*-quinone complexes been carried out. It has been reported that *o-*quinone derivatives are non-innocent electroactive ligands that can be found as neutral quinones, radical semi­quinones or dianionic catecholates (Lever *et al.*, 1988 ▸). The coordination chemistry of ruthenium complexes has been studied over the past few decades because of their versatile and diverse applications in mol­ecular catalysis (Pagliaro *et al.*, 2005 ▸; Ramakrishna & Bhat, 2011 ▸) and bioinorganic chemistry (van Rijt & Sadler, 2009 ▸). Ruthenium complexes with two *o*-quinone derivatives and one 2,2′-bi­pyridine (bpy) ligand, namely [Ru(bpy)(C_6_H_4_O_2_)_2_] and [Ru(bpy)(C_14_H_20_O_2_)_2_] (title compound), have been investigated by using various experimental techniques (Lever *et al.*, 1988 ▸). Although the ruthenium metals in these complexes potentially could be in the (II), (III) or (IV) oxidation state, according to the oxidation states of the two *o*-quinone ligands, the state of the metals was confirmed to be bivalent by photoelectron spectroscopy. In order to obtain ruthenium(III) species, it was necessary to oxidize the complexes by silver perchlorate in non-aqueous media. Lever *et al.* (1988 ▸) concluded that the complexes are best regarded as Ru^II^(bpy)(sq)_2_ (sq: semi­quinone anion-radical) with significant mixing of metal and ligand orbitals through Ru–sq π back-donation, which results in elongation of the C—O bonds of *o*-quinone ligands. This elongation has been demonstrated for [Ru(bpy)(C_6_H_4_O_2_)_2_] by X-ray single crystal analysis, but the structure of the title compound has not previously been characterized.
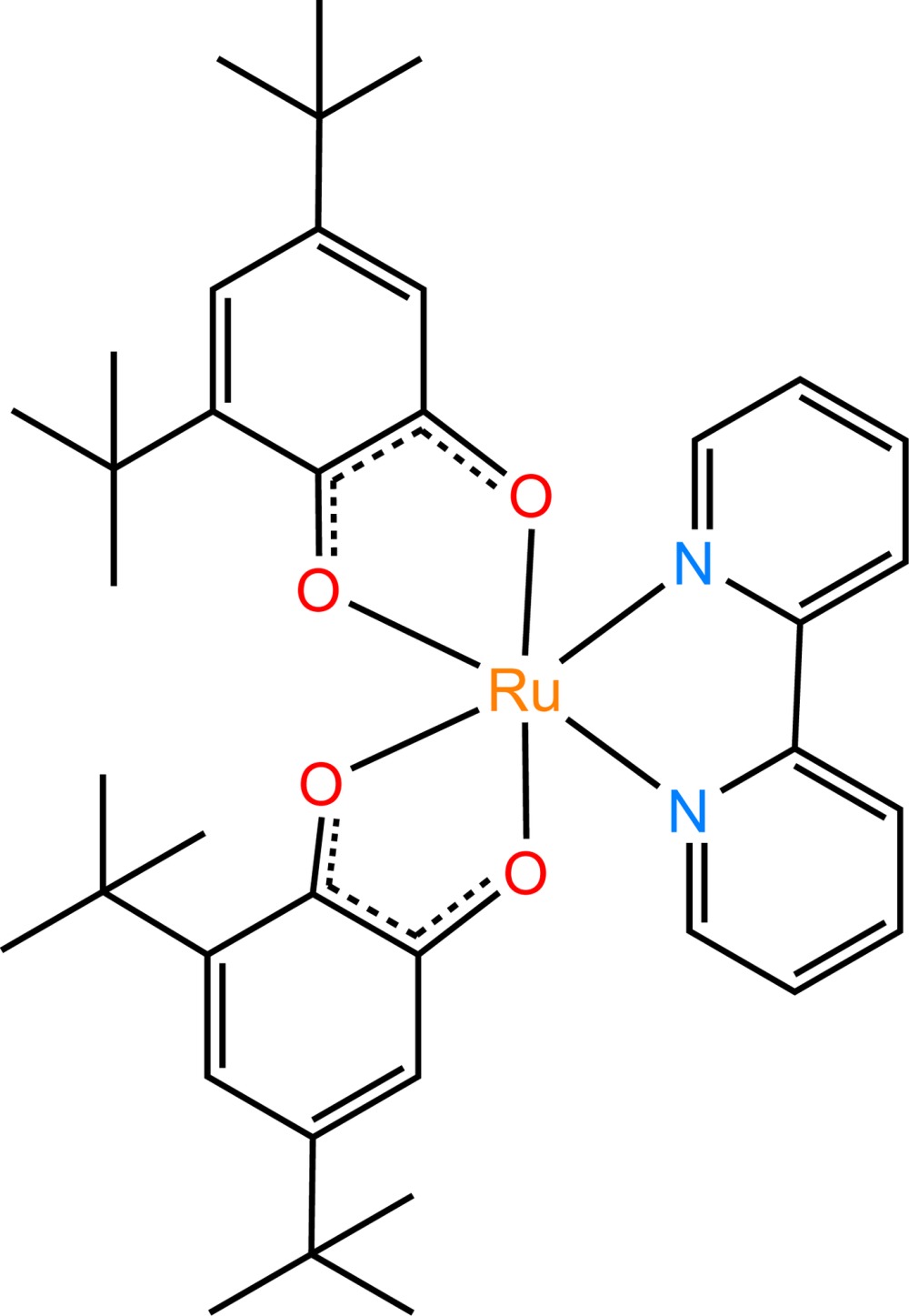


## Structural commentary   

**Figure 1:**
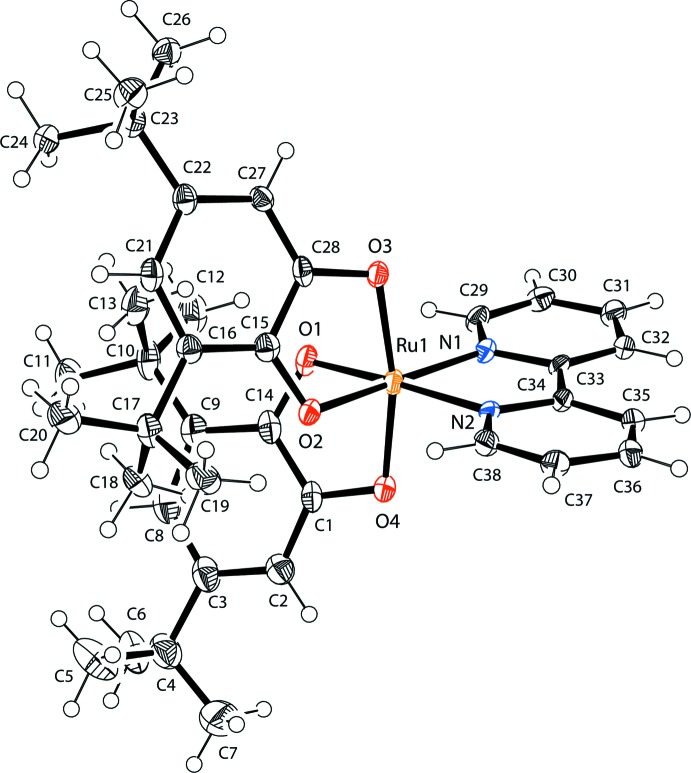
The mol­ecular structure of the title compound with the atom-labelling scheme. Displacement ellipsoids are drawn at the 40% probability level.

In the title compound, the Ru^II^ ion has a distorted octa­hedral RuN_2_O_4_ coordination environment defined by two 3,5-di-*tert*-butyl-*o-*benzosemi­quinone anion-radicals and one 2,2′-bi­pyridine ligand (Fig. 1 ▸). The coordination environment is identified by Ru—O and Ru—N bonds (Table 1 ▸). The C—N and C—C bond lengths in the 2,2′-bi­pyridine ligand are normal for 2-substituted pyridine derivatives (Krämer & Fritsky, 2000 ▸; Strotmeyer *et al.*, 2003 ▸; Moroz *et al.*, 2012 ▸). The benzosemi­quinone ligands exhibit almost equivalent C—O distances (Table 1 ▸). These bond lengths are inter­mediate between values expected for the semi­quinone (1.29 Å) and catecholate (1.34 Å) forms (Buchanan *et al.*, 1978 ▸). The Ru—O, Ru—N, C—O and C—C bond lengths in the title complex are very close to those observed in [Ru(bpy)(C_6_H_4_O_2_)_2_] (Lever *et al.*, 1988 ▸).

## Supra­molecular features   

**Figure 2:**
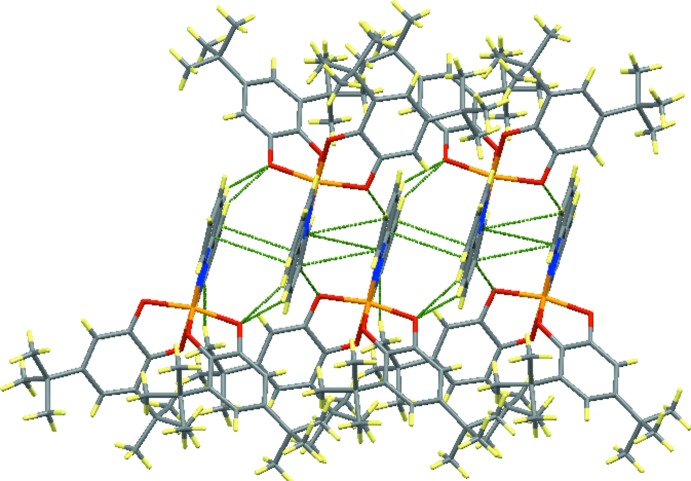
A packing view of the title compound with the C—H⋯O and π–π inter­actions shown as dashed lines.

In the crystal, the complex mol­ecules are linked *via* C—H⋯O hydrogen bonds (Table 2 ▸) and π–π stacking inter­actions between inversion-related 2,2′-bi­pyridine ligands [centroid–centroid distance = 3.538 (3) Å], which results in a layer structure parallel to the *ab* plane (Figs. 2 ▸ and 3 ▸).

## Database survey   

A search of the Cambridge Structural Database (CSD, Version 5.37, update May 2016; Groom *et al.*, 2016 ▸) gave 14 hits for mononuclear ruthenium complexes with 3,5-di-*tert*-butyl-*o-*benzo­quinone ligands in three possible catecholate, semi­quinone and quinone forms (CSD refcodes: EHUMEZ, EHUMID, EHUMOJ, FAGKON, FAGKON10, FIHQOC, FIRVIL, JECHII, JECHOO, MAFHOR, SAHHUF, SOCCAO, VINZIB, WUPGUJ).

## Synthesis and crystallization   

3,5-Di-*tert*-butyl-*o*-benzo­quinone (0.2 g, 0.90 mmol) was added to 20 ml dry methanol and then tri­ethyl­amine (0.181 g, 1.8 mmol) was added dropwise and the resultant mixture was stirred for 5 min. Ru(bpy)_2_Cl_2_ (0.288 g, 0.45 mmol) was then added to the solution and the contents were refluxed for 6 h. After refluxing, the reaction mixture was cooled down to room temperature and the contents were filtered off. The obtained residue was washed with cold methanol and dried *in vacuo* (yield: 0.160 g, 70%). Slow evaporation of a solution of the compound in a CH_2_Cl_2_–MeOH mixture (1:1, *v*/*v*) yielded single crystals suitable for X-ray diffraction. Crystals of title compound gave no EPR signal at room and liquid nitro­gen temperatures, and thus are diamagnetic.

## Refinement   

Crystal data, data collection and structure refinement details are summarized in Table 3 ▸. H atoms of the methyl groups were located in a difference Fourier map and refined as part of rigid rotating groups, with C—H = 0.96 Å and *U*_iso_(H) = 1.5*U*_eq_(C). The remaining (aromatic) H atoms were placed geometrically and refined using a riding model, with C—H = 0.93 Å and *U*_iso_(H) = 1.2*U*_eq_(C).

## Supplementary Material

Crystal structure: contains datablock(s) I. DOI: 10.1107/S205698901700281X/is5467sup1.cif

Structure factors: contains datablock(s) I. DOI: 10.1107/S205698901700281X/is5467Isup2.hkl

CCDC reference: 1533648

Additional supporting information:  crystallographic information; 3D view; checkCIF report

## Figures and Tables

**Figure 3:**
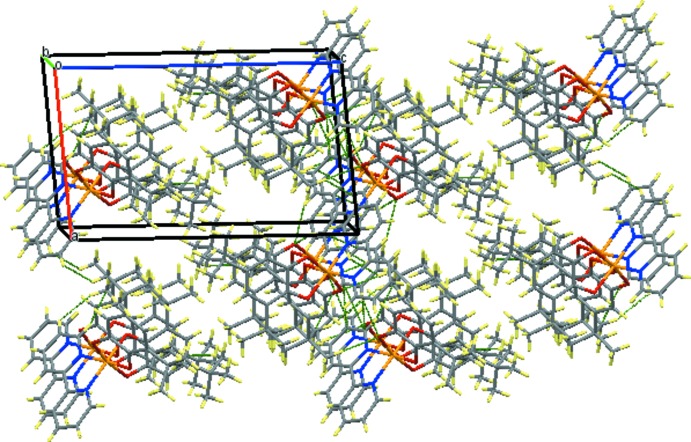
A packing diagram of the title compound viewed along the *b* axis.

**Table 1:** Selected bond lengths (Å)

Ru1—O2	1.978 (3)	Ru1—N1	2.053 (4)
Ru1—O4	1.988 (3)	O1—C14	1.340 (6)
Ru1—O1	1.990 (3)	O2—C15	1.325 (5)
Ru1—O3	1.994 (3)	O3—C28	1.340 (5)
Ru1—N2	2.044 (4)	O4—C1	1.332 (6)

**Table 2:** Hydrogen-bond geometry (Å, °)

*D*—H⋯*A*	*D*—H	H⋯*A*	*D*⋯*A*	*D*—H⋯*A*
C30—H30⋯O3^i^	0.93	2.54	3.427 (6)	159
C32—H32⋯O4^ii^	0.93	2.49	3.322 (6)	148
C35—H35⋯O4^ii^	0.93	2.39	3.232 (6)	151

Symmetry codes: (i) 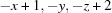; (ii) 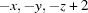.

**Table 3:** Experimental details

Crystal data	
Chemical formula	[Ru(C_14_H_20_O_2_)_2_(C_10_H_8_N_2_)]
*M*_r_	697.85
Crystal system, space group	Triclinic, *P* 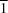
Temperature (K)	100
*a*, *b*, *c* (Å)	10.125 (5), 10.325 (5), 17.419 (5)
α, β, γ (°)	76.583 (5), 83.238 (5), 85.777 (5)
*V* (Å^3^)	1756.9 (13)
*Z*	2
Radiation type	Mo *K*α
μ (mm^−1^)	0.49
Crystal size (mm)	0.21 × 0.17 × 0.13

Data collection	
Diffractometer	Bruker SMART APEX CCD diffractometer
Absorption correction	Multi-scan (*SADABS*; Sheldrick, 2004 ▸)
*T*_min_, *T*_max_	0.902, 0.925
No. of measured, independent and observed [*I* > 2σ(*I*)] reflections	8836, 6058, 4508
*R*_int_	0.033
(sin θ/λ)_max_ (Å^−1^)	0.596

Refinement	
*R*[*F* ^2^ > 2σ(*F* ^2^)], *wR*(*F* ^2^), *S*	0.051, 0.142, 1.02
No. of reflections	6058
No. of parameters	418
H-atom treatment	H-atom parameters constrained
Δρ_max_, Δρ_min_ (e Å^−3^)	0.95, −0.48

Computer programs: *SMART* and *SAINT* (Bruker, 2003 ▸), *SIR97* (Altomare *et al.*, 1999 ▸), *SHELXL2014/6* (Sheldrick, 2015 ▸) and *DIAMOND* (Brandenberg & Putz, 2006 ▸).

## supplementary crystallographic information

### Crystal data

**Table:**

[Ru(C_14_H_20_O_2_)_2_(C_10_H_8_N_2_)]	*Z* = 2
*M**_r_* = 697.85	*F*(000) = 732
Triclinic, *P*1	*D*_x_ = 1.319 Mg m^−^^3^
*a* = 10.125 (5) Å	Mo *K*α radiation, λ = 0.71073 Å
*b* = 10.325 (5) Å	Cell parameters from 1535 reflections
*c* = 17.419 (5) Å	θ = 2.0–25.0°
α = 76.583 (5)°	µ = 0.49 mm^−^^1^
β = 83.238 (5)°	*T* = 100 K
γ = 85.777 (5)°	Block, red
*V* = 1756.9 (13) Å^3^	0.21 × 0.17 × 0.13 mm

### Data collection

**Table:**

Bruker SMART APEX CCD diffractometer	6058 independent reflections
Radiation source: fine-focus sealed tube	4508 reflections with *I* > 2σ(*I*)
Graphite monochromator	*R*_int_ = 0.033
/w–scans	θ_max_ = 25.1°, θ_min_ = 2.0°
Absorption correction: multi-scan (SADABS; Sheldrick, 2004)	*h* = −12→9
*T*_min_ = 0.902, *T*_max_ = 0.925	*k* = −12→12
8836 measured reflections	*l* = −20→20

### Refinement

**Table:**

Refinement on *F*^2^	0 restraints
Least-squares matrix: full	Hydrogen site location: inferred from neighbouring sites
*R*[*F*^2^ > 2σ(*F*^2^)] = 0.051	H-atom parameters constrained
*wR*(*F*^2^) = 0.142	*w* = 1/[σ^2^(*F*_o_^2^) + (0.0802*P*)^2^] where *P* = (*F*_o_^2^ + 2*F*_c_^2^)/3
*S* = 1.02	(Δ/σ)_max_ = 0.001
6058 reflections	Δρ_max_ = 0.95 e Å^−^^3^
418 parameters	Δρ_min_ = −0.48 e Å^−^^3^

### Special details

**Table:**

Geometry. All esds (except the esd in the dihedral angle between two l.s. planes) are estimated using the full covariance matrix. The cell esds are taken into account individually in the estimation of esds in distances, angles and torsion angles; correlations between esds in cell parameters are only used when they are defined by crystal symmetry. An approximate (isotropic) treatment of cell esds is used for estimating esds involving l.s. planes.

### Fractional atomic coordinates and isotropic or equivalent isotropic displacement parameters (Å^2^)

**Table:**

	*x*	*y*	*z*	*U*_iso_*/*U*_eq_	
Ru1	0.23460 (4)	0.24040 (4)	0.88962 (2)	0.02318 (15)	
O1	0.3873 (3)	0.2156 (3)	0.81046 (19)	0.0282 (8)	
O2	0.2021 (3)	0.4171 (3)	0.81903 (18)	0.0248 (7)	
O3	0.3497 (3)	0.3434 (3)	0.93673 (17)	0.0230 (7)	
O4	0.1382 (3)	0.1589 (3)	0.82104 (18)	0.0265 (7)	
N1	0.2578 (4)	0.0644 (4)	0.9718 (2)	0.0218 (8)	
N2	0.0725 (3)	0.2515 (4)	0.9701 (2)	0.0195 (8)	
C1	0.2025 (5)	0.1849 (5)	0.7483 (3)	0.0302 (12)	
C2	0.1427 (6)	0.1768 (5)	0.6815 (3)	0.0345 (12)	
H2	0.0544	0.1539	0.6868	0.041*	
C3	0.2142 (6)	0.2025 (5)	0.6076 (3)	0.0405 (14)	
C4	0.1509 (6)	0.2096 (6)	0.5313 (3)	0.0476 (15)	
C5	0.1523 (8)	0.3538 (7)	0.4812 (4)	0.071 (2)	
H5A	0.1063	0.4127	0.5119	0.107*	
H5B	0.1087	0.3594	0.4344	0.107*	
H5C	0.2427	0.3792	0.4662	0.107*	
C6	0.2347 (7)	0.1222 (6)	0.4809 (3)	0.0571 (18)	
H6A	0.3219	0.1562	0.4657	0.086*	
H6B	0.1922	0.1238	0.4343	0.086*	
H6C	0.2419	0.0322	0.5115	0.086*	
C7	0.0131 (8)	0.1589 (9)	0.5479 (4)	0.085 (3)	
H7A	0.0148	0.0718	0.5826	0.127*	
H7B	−0.0190	0.1540	0.4990	0.127*	
H7C	−0.0449	0.2184	0.5728	0.127*	
C8	0.3507 (6)	0.2296 (6)	0.6035 (3)	0.0430 (14)	
H8	0.3991	0.2444	0.5537	0.052*	
C9	0.4180 (6)	0.2358 (5)	0.6674 (3)	0.0369 (13)	
C10	0.5641 (6)	0.2666 (6)	0.6581 (3)	0.0422 (14)	
C11	0.6255 (6)	0.2910 (7)	0.5711 (3)	0.0560 (17)	
H11A	0.7175	0.3104	0.5681	0.084*	
H11B	0.5785	0.3651	0.5399	0.084*	
H11C	0.6187	0.2128	0.5511	0.084*	
C12	0.6420 (6)	0.1452 (6)	0.7054 (4)	0.0503 (16)	
H12A	0.6319	0.0681	0.6850	0.075*	
H12B	0.6078	0.1287	0.7604	0.075*	
H12C	0.7347	0.1636	0.7001	0.075*	
C13	0.5878 (6)	0.3889 (6)	0.6898 (3)	0.0480 (15)	
H13A	0.6813	0.4040	0.6829	0.072*	
H13B	0.5562	0.3735	0.7452	0.072*	
H13C	0.5407	0.4657	0.6612	0.072*	
C14	0.3400 (5)	0.2136 (5)	0.7421 (3)	0.0330 (12)	
C15	0.3032 (5)	0.4926 (5)	0.8173 (3)	0.0268 (11)	
C16	0.3288 (5)	0.6132 (5)	0.7597 (3)	0.0265 (11)	
C17	0.2379 (5)	0.6627 (5)	0.6931 (3)	0.0332 (12)	
C18	0.2382 (6)	0.5619 (6)	0.6419 (3)	0.0406 (14)	
H18A	0.3282	0.5418	0.6222	0.061*	
H18B	0.1872	0.5984	0.5981	0.061*	
H18C	0.1996	0.4817	0.6731	0.061*	
C19	0.0951 (5)	0.6859 (6)	0.7286 (3)	0.0415 (14)	
H19A	0.0667	0.6064	0.7659	0.062*	
H19B	0.0374	0.7078	0.6869	0.062*	
H19C	0.0913	0.7580	0.7551	0.062*	
C20	0.2839 (6)	0.7960 (6)	0.6391 (3)	0.0480 (16)	
H20A	0.2800	0.8624	0.6700	0.072*	
H20B	0.2265	0.8244	0.5976	0.072*	
H20C	0.3737	0.7842	0.6162	0.072*	
C21	0.4410 (5)	0.6772 (5)	0.7656 (3)	0.0285 (11)	
H21	0.4591	0.7565	0.7287	0.034*	
C22	0.5310 (5)	0.6303 (5)	0.8242 (3)	0.0267 (11)	
C23	0.6572 (5)	0.7058 (5)	0.8201 (3)	0.0293 (11)	
C24	0.7223 (5)	0.7433 (6)	0.7339 (3)	0.0377 (13)	
H24A	0.8030	0.7876	0.7323	0.057*	
H24B	0.6621	0.8018	0.7015	0.057*	
H24C	0.7424	0.6640	0.7140	0.057*	
C25	0.6181 (6)	0.8344 (5)	0.8485 (3)	0.0395 (13)	
H25A	0.5891	0.8128	0.9043	0.059*	
H25B	0.5470	0.8818	0.8201	0.059*	
H25C	0.6935	0.8892	0.8390	0.059*	
C26	0.7603 (5)	0.6276 (5)	0.8714 (3)	0.0350 (12)	
H26A	0.7264	0.6151	0.9263	0.052*	
H26B	0.8403	0.6759	0.8619	0.052*	
H26C	0.7795	0.5423	0.8583	0.052*	
C27	0.5004 (5)	0.5190 (4)	0.8824 (3)	0.0234 (10)	
H27	0.5543	0.4899	0.9234	0.028*	
C28	0.3879 (5)	0.4494 (5)	0.8800 (3)	0.0263 (11)	
C29	0.3592 (4)	−0.0251 (5)	0.9692 (3)	0.0259 (11)	
H29	0.4255	−0.0085	0.9268	0.031*	
C30	0.3697 (5)	−0.1417 (5)	1.0271 (3)	0.0298 (11)	
H30	0.4428	−0.2010	1.0239	0.036*	
C31	0.2708 (5)	−0.1692 (5)	1.0893 (3)	0.0290 (11)	
H31	0.2759	−0.2474	1.1284	0.035*	
C32	0.1639 (5)	−0.0781 (5)	1.0924 (3)	0.0245 (11)	
H32	0.0957	−0.0949	1.1335	0.029*	
C33	0.1593 (4)	0.0378 (4)	1.0338 (3)	0.0210 (10)	
C34	0.0541 (4)	0.1448 (4)	1.0321 (3)	0.0202 (10)	
C35	−0.0556 (5)	0.1385 (5)	1.0880 (3)	0.0250 (11)	
H35	−0.0676	0.0632	1.1290	0.030*	
C36	−0.1470 (5)	0.2455 (5)	1.0822 (3)	0.0278 (11)	
H36	−0.2210	0.2435	1.1195	0.033*	
C37	−0.1272 (5)	0.3559 (5)	1.0201 (3)	0.0267 (11)	
H37	−0.1869	0.4295	1.0157	0.032*	
C38	−0.0182 (5)	0.3549 (5)	0.9653 (3)	0.0265 (11)	
H38	−0.0064	0.4285	0.9231	0.032*	

### Atomic displacement parameters (Å^2^)

**Table:**

	*U*^11^	*U*^22^	*U*^33^	*U*^12^	*U*^13^	*U*^23^
Ru1	0.0207 (2)	0.0254 (2)	0.0202 (2)	−0.00348 (15)	0.00222 (14)	0.00001 (15)
O1	0.0239 (18)	0.0285 (18)	0.0270 (19)	−0.0015 (15)	0.0055 (14)	0.0005 (15)
O2	0.0191 (17)	0.0296 (18)	0.0230 (18)	−0.0046 (14)	0.0020 (13)	−0.0014 (14)
O3	0.0200 (17)	0.0250 (17)	0.0208 (17)	−0.0023 (14)	0.0014 (13)	−0.0002 (14)
O4	0.0244 (18)	0.0298 (18)	0.0229 (18)	−0.0054 (15)	0.0021 (14)	−0.0019 (14)
N1	0.021 (2)	0.020 (2)	0.023 (2)	−0.0054 (17)	0.0011 (16)	−0.0031 (17)
N2	0.015 (2)	0.022 (2)	0.020 (2)	−0.0029 (16)	−0.0050 (15)	0.0014 (16)
C1	0.037 (3)	0.028 (3)	0.022 (3)	−0.006 (2)	0.004 (2)	0.000 (2)
C2	0.041 (3)	0.031 (3)	0.032 (3)	−0.003 (2)	−0.004 (2)	−0.007 (2)
C3	0.055 (4)	0.037 (3)	0.029 (3)	−0.007 (3)	0.001 (3)	−0.009 (3)
C4	0.061 (4)	0.051 (4)	0.033 (3)	−0.008 (3)	−0.005 (3)	−0.011 (3)
C5	0.099 (6)	0.068 (5)	0.050 (4)	0.016 (4)	−0.030 (4)	−0.012 (4)
C6	0.088 (5)	0.044 (4)	0.035 (4)	−0.007 (4)	−0.002 (3)	0.000 (3)
C7	0.071 (5)	0.143 (8)	0.048 (4)	−0.032 (5)	−0.007 (4)	−0.026 (5)
C8	0.052 (4)	0.043 (3)	0.030 (3)	−0.003 (3)	0.007 (3)	−0.007 (3)
C9	0.041 (3)	0.037 (3)	0.031 (3)	−0.001 (3)	0.003 (2)	−0.006 (2)
C10	0.046 (4)	0.041 (3)	0.031 (3)	0.002 (3)	0.017 (3)	−0.003 (3)
C11	0.043 (4)	0.079 (5)	0.034 (3)	0.008 (3)	0.012 (3)	−0.001 (3)
C12	0.044 (4)	0.045 (4)	0.050 (4)	0.009 (3)	0.010 (3)	0.002 (3)
C13	0.042 (4)	0.052 (4)	0.044 (4)	−0.004 (3)	0.012 (3)	−0.005 (3)
C14	0.044 (3)	0.027 (3)	0.025 (3)	−0.004 (2)	0.003 (2)	−0.002 (2)
C15	0.025 (3)	0.027 (3)	0.026 (3)	0.003 (2)	0.001 (2)	−0.006 (2)
C16	0.027 (3)	0.024 (3)	0.026 (3)	0.003 (2)	−0.002 (2)	−0.002 (2)
C17	0.036 (3)	0.034 (3)	0.025 (3)	0.002 (2)	0.002 (2)	−0.001 (2)
C18	0.045 (3)	0.045 (3)	0.028 (3)	−0.003 (3)	−0.012 (2)	0.005 (3)
C19	0.031 (3)	0.055 (4)	0.035 (3)	0.012 (3)	−0.008 (2)	−0.006 (3)
C20	0.051 (4)	0.043 (3)	0.044 (4)	−0.005 (3)	−0.019 (3)	0.008 (3)
C21	0.031 (3)	0.022 (3)	0.027 (3)	0.000 (2)	0.004 (2)	0.000 (2)
C22	0.027 (3)	0.027 (3)	0.024 (3)	0.002 (2)	0.003 (2)	−0.004 (2)
C23	0.027 (3)	0.029 (3)	0.028 (3)	−0.005 (2)	0.003 (2)	−0.001 (2)
C24	0.028 (3)	0.054 (4)	0.029 (3)	−0.012 (3)	0.001 (2)	−0.003 (3)
C25	0.037 (3)	0.037 (3)	0.044 (3)	−0.005 (3)	−0.004 (3)	−0.008 (3)
C26	0.029 (3)	0.037 (3)	0.036 (3)	−0.001 (2)	−0.004 (2)	−0.001 (2)
C27	0.023 (3)	0.026 (3)	0.020 (2)	0.002 (2)	−0.0039 (19)	−0.002 (2)
C28	0.029 (3)	0.030 (3)	0.016 (2)	−0.001 (2)	0.0051 (19)	−0.002 (2)
C29	0.016 (2)	0.028 (3)	0.031 (3)	−0.001 (2)	0.002 (2)	−0.003 (2)
C30	0.023 (3)	0.027 (3)	0.040 (3)	−0.001 (2)	−0.006 (2)	−0.008 (2)
C31	0.030 (3)	0.020 (3)	0.034 (3)	−0.004 (2)	−0.006 (2)	0.004 (2)
C32	0.023 (3)	0.030 (3)	0.020 (3)	−0.007 (2)	−0.0008 (19)	−0.002 (2)
C33	0.017 (2)	0.025 (2)	0.022 (2)	−0.0087 (19)	0.0000 (18)	−0.005 (2)
C34	0.021 (2)	0.021 (2)	0.019 (2)	−0.0067 (19)	−0.0002 (18)	−0.003 (2)
C35	0.026 (3)	0.028 (3)	0.020 (3)	−0.004 (2)	−0.002 (2)	−0.002 (2)
C36	0.027 (3)	0.035 (3)	0.021 (3)	−0.002 (2)	0.000 (2)	−0.005 (2)
C37	0.021 (3)	0.026 (3)	0.032 (3)	0.005 (2)	−0.004 (2)	−0.006 (2)
C38	0.031 (3)	0.023 (3)	0.025 (3)	−0.006 (2)	−0.004 (2)	−0.003 (2)

### Geometric parameters (Å, º)

**Table:**

Ru1—O2	1.978 (3)	C16—C21	1.381 (6)
Ru1—O4	1.988 (3)	C16—C17	1.536 (7)
Ru1—O1	1.990 (3)	C17—C18	1.520 (7)
Ru1—O3	1.994 (3)	C17—C19	1.529 (7)
Ru1—N2	2.044 (4)	C17—C20	1.545 (7)
Ru1—N1	2.053 (4)	C18—H18A	0.9600
O1—C14	1.340 (6)	C18—H18B	0.9600
O2—C15	1.325 (5)	C18—H18C	0.9600
O3—C28	1.340 (5)	C19—H19A	0.9600
O4—C1	1.332 (6)	C19—H19B	0.9600
N1—C29	1.334 (6)	C19—H19C	0.9600
N1—C33	1.373 (6)	C20—H20A	0.9600
N2—C38	1.350 (6)	C20—H20B	0.9600
N2—C34	1.359 (5)	C20—H20C	0.9600
C1—C2	1.395 (7)	C21—C22	1.426 (7)
C1—C14	1.431 (7)	C21—H21	0.9300
C2—C3	1.379 (7)	C22—C27	1.372 (6)
C2—H2	0.9300	C22—C23	1.531 (7)
C3—C8	1.421 (8)	C23—C26	1.516 (7)
C3—C4	1.526 (7)	C23—C25	1.532 (7)
C4—C7	1.499 (9)	C23—C24	1.542 (7)
C4—C5	1.542 (9)	C24—H24A	0.9600
C4—C6	1.543 (9)	C24—H24B	0.9600
C5—H5A	0.9600	C24—H24C	0.9600
C5—H5B	0.9600	C25—H25A	0.9600
C5—H5C	0.9600	C25—H25B	0.9600
C6—H6A	0.9600	C25—H25C	0.9600
C6—H6B	0.9600	C26—H26A	0.9600
C6—H6C	0.9600	C26—H26B	0.9600
C7—H7A	0.9600	C26—H26C	0.9600
C7—H7B	0.9600	C27—C28	1.401 (6)
C7—H7C	0.9600	C27—H27	0.9300
C8—C9	1.387 (7)	C29—C30	1.386 (7)
C8—H8	0.9300	C29—H29	0.9300
C9—C14	1.421 (7)	C30—C31	1.379 (7)
C9—C10	1.518 (8)	C30—H30	0.9300
C10—C13	1.536 (8)	C31—C32	1.386 (7)
C10—C11	1.539 (7)	C31—H31	0.9300
C10—C12	1.550 (8)	C32—C33	1.382 (6)
C11—H11A	0.9600	C32—H32	0.9300
C11—H11B	0.9600	C33—C34	1.474 (6)
C11—H11C	0.9600	C34—C35	1.384 (6)
C12—H12A	0.9600	C35—C36	1.381 (7)
C12—H12B	0.9600	C35—H35	0.9300
C12—H12C	0.9600	C36—C37	1.385 (7)
C13—H13A	0.9600	C36—H36	0.9300
C13—H13B	0.9600	C37—C38	1.373 (7)
C13—H13C	0.9600	C37—H37	0.9300
C15—C16	1.425 (6)	C38—H38	0.9300
C15—C28	1.438 (6)		
			
O2—Ru1—O4	89.04 (13)	C21—C16—C15	116.4 (4)
O2—Ru1—O1	86.32 (13)	C21—C16—C17	123.2 (4)
O4—Ru1—O1	81.90 (13)	C15—C16—C17	120.3 (4)
O2—Ru1—O3	82.41 (12)	C18—C17—C19	108.6 (4)
O4—Ru1—O3	167.90 (12)	C18—C17—C16	111.1 (4)
O1—Ru1—O3	88.99 (13)	C19—C17—C16	110.1 (4)
O2—Ru1—N2	96.44 (14)	C18—C17—C20	108.2 (4)
O4—Ru1—N2	94.41 (13)	C19—C17—C20	107.9 (4)
O1—Ru1—N2	175.38 (14)	C16—C17—C20	110.9 (4)
O3—Ru1—N2	95.04 (13)	C17—C18—H18A	109.5
O2—Ru1—N1	174.28 (14)	C17—C18—H18B	109.5
O4—Ru1—N1	93.75 (14)	H18A—C18—H18B	109.5
O1—Ru1—N1	99.00 (14)	C17—C18—H18C	109.5
O3—Ru1—N1	95.53 (13)	H18A—C18—H18C	109.5
N2—Ru1—N1	78.39 (15)	H18B—C18—H18C	109.5
C14—O1—Ru1	108.6 (3)	C17—C19—H19A	109.5
C15—O2—Ru1	109.2 (3)	C17—C19—H19B	109.5
C28—O3—Ru1	107.3 (3)	H19A—C19—H19B	109.5
C1—O4—Ru1	108.0 (3)	C17—C19—H19C	109.5
C29—N1—C33	118.2 (4)	H19A—C19—H19C	109.5
C29—N1—Ru1	125.3 (3)	H19B—C19—H19C	109.5
C33—N1—Ru1	116.5 (3)	C17—C20—H20A	109.5
C38—N2—C34	118.0 (4)	C17—C20—H20B	109.5
C38—N2—Ru1	125.1 (3)	H20A—C20—H20B	109.5
C34—N2—Ru1	116.9 (3)	C17—C20—H20C	109.5
O4—C1—C2	122.4 (5)	H20A—C20—H20C	109.5
O4—C1—C14	116.6 (4)	H20B—C20—H20C	109.5
C2—C1—C14	120.9 (5)	C16—C21—C22	124.5 (4)
C3—C2—C1	120.2 (5)	C16—C21—H21	117.7
C3—C2—H2	119.9	C22—C21—H21	117.7
C1—C2—H2	119.9	C27—C22—C21	118.4 (4)
C2—C3—C8	117.5 (5)	C27—C22—C23	122.6 (4)
C2—C3—C4	122.8 (5)	C21—C22—C23	119.0 (4)
C8—C3—C4	119.7 (5)	C26—C23—C22	113.5 (4)
C7—C4—C3	111.8 (5)	C26—C23—C25	108.0 (4)
C7—C4—C5	111.7 (6)	C22—C23—C25	108.4 (4)
C3—C4—C5	109.3 (5)	C26—C23—C24	108.1 (4)
C7—C4—C6	107.2 (6)	C22—C23—C24	110.2 (4)
C3—C4—C6	109.9 (5)	C25—C23—C24	108.4 (4)
C5—C4—C6	106.7 (5)	C23—C24—H24A	109.5
C4—C5—H5A	109.5	C23—C24—H24B	109.5
C4—C5—H5B	109.5	H24A—C24—H24B	109.5
H5A—C5—H5B	109.5	C23—C24—H24C	109.5
C4—C5—H5C	109.5	H24A—C24—H24C	109.5
H5A—C5—H5C	109.5	H24B—C24—H24C	109.5
H5B—C5—H5C	109.5	C23—C25—H25A	109.5
C4—C6—H6A	109.5	C23—C25—H25B	109.5
C4—C6—H6B	109.5	H25A—C25—H25B	109.5
H6A—C6—H6B	109.5	C23—C25—H25C	109.5
C4—C6—H6C	109.5	H25A—C25—H25C	109.5
H6A—C6—H6C	109.5	H25B—C25—H25C	109.5
H6B—C6—H6C	109.5	C23—C26—H26A	109.5
C4—C7—H7A	109.5	C23—C26—H26B	109.5
C4—C7—H7B	109.5	H26A—C26—H26B	109.5
H7A—C7—H7B	109.5	C23—C26—H26C	109.5
C4—C7—H7C	109.5	H26A—C26—H26C	109.5
H7A—C7—H7C	109.5	H26B—C26—H26C	109.5
H7B—C7—H7C	109.5	C22—C27—C28	119.8 (4)
C9—C8—C3	125.6 (5)	C22—C27—H27	120.1
C9—C8—H8	117.2	C28—C27—H27	120.1
C3—C8—H8	117.2	O3—C28—C27	122.7 (4)
C8—C9—C14	115.3 (5)	O3—C28—C15	116.2 (4)
C8—C9—C10	122.5 (5)	C27—C28—C15	121.1 (4)
C14—C9—C10	122.2 (5)	N1—C29—C30	122.5 (4)
C9—C10—C13	112.5 (5)	N1—C29—H29	118.7
C9—C10—C11	112.4 (5)	C30—C29—H29	118.7
C13—C10—C11	107.8 (5)	C31—C30—C29	119.5 (5)
C9—C10—C12	108.7 (5)	C31—C30—H30	120.3
C13—C10—C12	107.9 (5)	C29—C30—H30	120.3
C11—C10—C12	107.3 (5)	C30—C31—C32	118.7 (5)
C10—C11—H11A	109.5	C30—C31—H31	120.7
C10—C11—H11B	109.5	C32—C31—H31	120.7
H11A—C11—H11B	109.5	C33—C32—C31	119.6 (4)
C10—C11—H11C	109.5	C33—C32—H32	120.2
H11A—C11—H11C	109.5	C31—C32—H32	120.2
H11B—C11—H11C	109.5	N1—C33—C32	121.5 (4)
C10—C12—H12A	109.5	N1—C33—C34	113.6 (4)
C10—C12—H12B	109.5	C32—C33—C34	124.8 (4)
H12A—C12—H12B	109.5	N2—C34—C35	121.9 (4)
C10—C12—H12C	109.5	N2—C34—C33	114.5 (4)
H12A—C12—H12C	109.5	C35—C34—C33	123.6 (4)
H12B—C12—H12C	109.5	C36—C35—C34	119.1 (4)
C10—C13—H13A	109.5	C36—C35—H35	120.4
C10—C13—H13B	109.5	C34—C35—H35	120.4
H13A—C13—H13B	109.5	C35—C36—C37	119.2 (5)
C10—C13—H13C	109.5	C35—C36—H36	120.4
H13A—C13—H13C	109.5	C37—C36—H36	120.4
H13B—C13—H13C	109.5	C38—C37—C36	119.0 (4)
O1—C14—C9	124.0 (5)	C38—C37—H37	120.5
O1—C14—C1	115.6 (4)	C36—C37—H37	120.5
C9—C14—C1	120.4 (5)	N2—C38—C37	122.7 (4)
O2—C15—C16	124.3 (4)	N2—C38—H38	118.7
O2—C15—C28	116.2 (4)	C37—C38—H38	118.7
C16—C15—C28	119.5 (4)		
			
Ru1—O4—C1—C2	−160.9 (4)	C17—C16—C21—C22	−176.7 (5)
Ru1—O4—C1—C14	23.0 (5)	C16—C21—C22—C27	−4.8 (7)
O4—C1—C2—C3	−178.9 (5)	C16—C21—C22—C23	175.5 (5)
C14—C1—C2—C3	−3.0 (8)	C27—C22—C23—C26	15.3 (7)
C1—C2—C3—C8	3.5 (8)	C21—C22—C23—C26	−165.0 (4)
C1—C2—C3—C4	−173.5 (5)	C27—C22—C23—C25	−104.6 (5)
C2—C3—C4—C7	−11.4 (9)	C21—C22—C23—C25	75.1 (6)
C8—C3—C4—C7	171.7 (6)	C27—C22—C23—C24	136.8 (5)
C2—C3—C4—C5	112.8 (6)	C21—C22—C23—C24	−43.5 (6)
C8—C3—C4—C5	−64.1 (7)	C21—C22—C27—C28	4.4 (7)
C2—C3—C4—C6	−130.4 (6)	C23—C22—C27—C28	−175.9 (4)
C8—C3—C4—C6	52.7 (7)	Ru1—O3—C28—C27	−158.8 (4)
C2—C3—C8—C9	−1.8 (9)	Ru1—O3—C28—C15	23.9 (5)
C4—C3—C8—C9	175.3 (6)	C22—C27—C28—O3	−177.2 (4)
C3—C8—C9—C14	−0.5 (8)	C22—C27—C28—C15	0.0 (7)
C3—C8—C9—C10	−179.4 (5)	O2—C15—C28—O3	−4.9 (6)
C8—C9—C10—C13	122.6 (6)	C16—C15—C28—O3	173.0 (4)
C14—C9—C10—C13	−56.3 (7)	O2—C15—C28—C27	177.7 (4)
C8—C9—C10—C11	0.7 (8)	C16—C15—C28—C27	−4.4 (7)
C14—C9—C10—C11	−178.2 (5)	C33—N1—C29—C30	−1.2 (6)
C8—C9—C10—C12	−117.9 (6)	Ru1—N1—C29—C30	179.4 (3)
C14—C9—C10—C12	63.2 (7)	N1—C29—C30—C31	1.4 (7)
Ru1—O1—C14—C9	161.2 (4)	C29—C30—C31—C32	−0.5 (7)
Ru1—O1—C14—C1	−19.8 (5)	C30—C31—C32—C33	−0.5 (7)
C8—C9—C14—O1	180.0 (5)	C29—N1—C33—C32	0.1 (6)
C10—C9—C14—O1	−1.0 (8)	Ru1—N1—C33—C32	179.5 (3)
C8—C9—C14—C1	1.0 (8)	C29—N1—C33—C34	178.4 (4)
C10—C9—C14—C1	180.0 (5)	Ru1—N1—C33—C34	−2.1 (5)
O4—C1—C14—O1	−2.3 (7)	C31—C32—C33—N1	0.8 (6)
C2—C1—C14—O1	−178.4 (4)	C31—C32—C33—C34	−177.4 (4)
O4—C1—C14—C9	176.8 (4)	C38—N2—C34—C35	1.8 (6)
C2—C1—C14—C9	0.7 (8)	Ru1—N2—C34—C35	−176.8 (3)
Ru1—O2—C15—C16	165.0 (4)	C38—N2—C34—C33	−178.7 (4)
Ru1—O2—C15—C28	−17.2 (5)	Ru1—N2—C34—C33	2.7 (5)
O2—C15—C16—C21	−178.2 (4)	N1—C33—C34—N2	−0.4 (5)
C28—C15—C16—C21	4.0 (7)	C32—C33—C34—N2	177.9 (4)
O2—C15—C16—C17	−1.0 (7)	N1—C33—C34—C35	179.1 (4)
C28—C15—C16—C17	−178.8 (4)	C32—C33—C34—C35	−2.6 (7)
C21—C16—C17—C18	116.4 (5)	N2—C34—C35—C36	−2.1 (6)
C15—C16—C17—C18	−60.6 (6)	C33—C34—C35—C36	178.5 (4)
C21—C16—C17—C19	−123.2 (5)	C34—C35—C36—C37	0.6 (7)
C15—C16—C17—C19	59.8 (6)	C35—C36—C37—C38	1.1 (7)
C21—C16—C17—C20	−4.0 (7)	C34—N2—C38—C37	−0.1 (6)
C15—C16—C17—C20	179.0 (5)	Ru1—N2—C38—C37	178.4 (3)
C15—C16—C21—C22	0.4 (7)	C36—C37—C38—N2	−1.3 (7)

### Hydrogen-bond geometry (Å, º)

**Table:**

*D*—H···*A*	*D*—H	H···*A*	*D*···*A*	*D*—H···*A*
C30—H30···O3^i^	0.93	2.54	3.427 (6)	159
C32—H32···O4^ii^	0.93	2.49	3.322 (6)	148
C35—H35···O4^ii^	0.93	2.39	3.232 (6)	151

Symmetry codes: (i) −*x*+1, −*y*, −*z*+2; (ii) −*x*, −*y*, −*z*+2.
